# Predicted strain coverage of a meningococcal multicomponent vaccine (4CMenB) in Portugal

**DOI:** 10.1371/journal.pone.0176177

**Published:** 2017-05-01

**Authors:** Maria João Simões, Célia Bettencourt, Rosita De Paola, Maria Giuliani, Mariagrazia Pizza, Monica Moschioni, Jorge Machado

**Affiliations:** 1 Department of Infectious Diseases, National Institute of Health Dr. Ricardo Jorge, Lisboa, Portugal; 2 GSK Vaccines, Siena, Italy; RIVM, NETHERLANDS

## Abstract

**Objective:**

Although the incidence of meningococcal disease has been declining over the past decade in Portugal MenB meningococci is still an important cause of meningitis and sepsis. The aim of this study was to estimate the strain coverage of the 4CMenB vaccine in Portugal in order to support health policies for prevention and control of meningococcal disease.

**Methods:**

Since 2002 the clinical and laboratory notification of meningococcal disease is mandatory in Portugal. National database includes since then all confirmed cases notified to the reference laboratory or to the Directorate of Health. Strains included in this study were all the invasive MenB isolated from the 1^st^ July 2011 to the 30^th^ June 2015, sent to the reference laboratory. To predict the vaccine strain coverage of the 4CMenB the expression and cross-reactivity of the surface antigens fHbp, NadA, NHBA were assessed by the Meningococcal Antigen Typing System (MATS) whereas PorA typing was performed by sequencing. The presence of at least one antigen with a Relative Potency (RP) greater than its MATS-positive bactericidal threshold RP value or the presence of PorA VR2 = 4 was considered to be predictive for a strain to be covered by the 4CMenB vaccine.

**Results:**

The estimated 4CMenB strain coverage in Portugal was 67.9%. The percentage of strain coverage in each of the four epidemiological years ranged from 63.9% to 73.7%.

Strains covered by one antigen represent 32.1% of the total of isolates, 29.2% of strains were covered by two antigens and 6.6% by three antigens. No strain had all the four antigens. Antigens that most contributed for coverage were NHBA and fHbp.

Data from Portugal is in accordance with the MATS predicted strain coverage in five European countries (England and Wales, France, Germany, Italy and Norway) that pointed to 78% coverage for strains collected in the epidemiological year 2007–2008.

## Introduction

*Neisseria meningitidis* is one of the major causes of bacterial invasive disease worldwide with an estimated 1.2 million cases per year and a mortality rate of about 11% [1]. From the 12 serogroups of *N*. *meningitidis* defined on the basis of their capsular polysaccharides biochemical structure [2] only five, serogroups A, B, C, W and Y, are commonly responsible for invasive disease [3]. Serogroup X has been causing disease in some African countries and rarely outside Africa [3].

The majority of cases reported by European countries to the European Centre for Disease Prevention and Control in 2012 correspond to serogroup B meningococci (MenB) [4]. In Portugal MenB has been also the most prevalent serogroup since 2003 and, from 2011 to 2015, the incidence rate ranged from 0.32/100,000 population in 2014 to 0.52/100,000 population in 2011 [5].

Vaccines are powerful tools in reducing the burden of disease. Unlike pure polysaccharide vaccines that display only short-term efficacy in children over two years of age and adults, the conjugate vaccines in which capsular polysaccharide is conjugated to an immunogenic protein (diphtheria or tetanus toxoid) induce immune memory and are immunogenic in children under two years of age [6]. This approach is not suitable for a MenB vaccine due to the lack of immunogenicity of serogroup B capsular polysaccharide [7]. In fact polysaccharide of serogroup B meningococci is a homopolymer of sialic acid residues and has structural similarities to human brain glycoproteins, therefore autoimmune disease could be triggered by its use in vaccines [7]. To overcome this challenge, research on vaccines against MenB has been focused on the outer membrane vesicles (OMV) which, although very effective, fails in providing broad protection against heterologous MenB strains due to the high variability of the outer membrane antigens, such as porins, present in OMV [8]. In recent years a genomic-based approach for the design of vaccines successfully resulted in the development of a four component vaccine that potentially provides broad protection against MenB, named 4CMenB (trade name Bexsero, GSK Vaccines). This vaccine contains four components: 3 protein antigens that are exposed on the surface of the bacterial cell and are able to elicit antibodies with bactericidal activity, Neisserial Heparin-Binding Antigen (NHBA), factor H binding protein (fHbp), Neisserial Adhesin A (NadA) and the OMV from the epidemic strain NZ98/254 of New Zealand, containing variant P1.4 of the major outer membrane protein PorA [9].

Considering the variability of Neisserial proteins the effectiveness of the 4CMenB vaccine depends on the level of antigen expression and the immune cross reactivity with antibodies induced by the vaccine antigens. To predict which MenB isolate in a given geographic area can be covered by the 4CMenB vaccine, the Meningococcal Antigen Typing System (MATS) was developed [9].

Although the incidence of meningococcal disease has been declining over the past decade in Portugal, namely after the introduction of MenC vaccine in the national immunization program in 2006, MenB meningococci is still an important cause of meningitis and sepsis. The aim of this study was to estimate the strain coverage of the 4CMenB vaccine in Portugal in order to support health policies for prevention and control of meningococcal disease (MD).

## Materials and methods

### Meningococcal isolates

Since 2002 a laboratory based surveillance system of invasive MD is ongoing in Portugal, becoming mandatory the clinical and laboratory notification of all cases. A laboratory network was implemented at the time, to accomplish that goal, which included all laboratories from hospitals with MD inpatients. These should send meningococcal isolates as well as culture negative clinical samples from suspected cases for lab confirmation by molecular methods and genotyping to the national reference laboratory of *Neisseria meningitidis* in the National Institute of Health Ricardo Jorge, Lisbon. The national database of MD includes since then all confirmed cases notified either to the reference laboratory or to the Directorate of Health. Strains included in this study were all the invasive MenB isolated in hospital laboratories from the 1^st^ July 2011 to the 30^th^ June 2015 that were sent to the reference laboratory and maintained viability during transport and storage. All strains were genotyped according to published protocols: serogroups were identified by PCR [10], serosubtypes were characterized by sequencing of the variable regions VR1 and VR2 of *porA* gene [10], the outer membrane protein FetA was characterized by sequencing the variable region of *fetA* gene [11] and multilocus sequence typing (MLST) was done [12] (S1 Table).

### MATS (Meningococcal Antigen Typing System)

To predict the vaccine strain coverage of the 4CMenB (fHbp, NadA, NHBA and PorA), the expression and cross-reactivity of the surface antigens fHbp, NadA, NHBA were assessed by the Meningococcal Antigen Typing System (MATS) at accredited VisMederi Laboratory in Siena (Italy), whereas PorA typing was performed at the National Institute of Health by Dr. Ricardo Jorge (Portugal) and at GSK Research Centre (Italy).

MATS combines three sandwich enzyme-linked immunosorbent assays (ELISA) for phenotypic expression of NHBA, fHbp, and NadA and the conventional sequencing of the PorA variable region (VR) 2, as described in Donnelly et al [9]. Briefly, the bacteria were grown on chocolate agar plates and then resuspended in Mueller-Hinton broth until an optical density (OD) at 600 nm (OD600) of 0.4 was reached. Following the addition of a detergent, the bacterial extracts were serially diluted in ELISA plates coated with an antigen-specific capture antibody. The binding of the antigen was then detected by means of a biotin-labeled secondary antibody and a streptavidin-horseradish peroxidase (HRP) conjugate. The plates were read at 492 nm in an ELISA reader. The results were analyzed with StatLIA (Brendan Technologies, Carlsbad, CA) by calculating the Relative Potency (RP) of the tested strain bacterial lysate compared to that of a reference strain that was treated identically and assayed in each microtiter plate (H44/76 for fHbp, NGH38 for NHBA, and 5/99 for NadA).

Predicted coverage using MATS positive bactericidal threshold (PBT) was calculated as described previously [9]. The presence of at least one antigen with a RP greater than its MATS-PBT relative potency value (0.012 for fHbp, 0.294 for NHBA and 0.009 for NadA) or the presence of PorA VR2 = 4 (matched to the OMV-NZ component of 4CMenB) was considered to be predictive for a strain to be covered by the 4CMenB vaccine. Strains that did not meet these criteria were considered not covered.

### Statistical analyses

Estimates of the 95% confidence intervals (95% CI) for the MATS-PBTs were derived on the basis of overall assay repeatability and reproducibility [13]. Chi-square test was used to assess the statistical significance of differences in strain coverage between epidemiological years and age groups.

## Results

The 106 studied isolates correspond to 57.9% of all MenB cases reported to the national database with date of onset from 1^st^ July 2011 to 30^th^ June 2015. The remaining cases correspond either to PCR confirmed cases (18.0%), cases not reported to the reference laboratory (12.6%) or strains that lost viability during transport or storage (11.5%).

The estimated 4CMenB strain coverage in Portugal was 67.9% (95% CI, 56–81%). The percentage of strain coverage in each of the four epidemiological year ranged, non significantly (p-value = 0.851), from 63.9% in 2014–2015 to 73.7% in 2013–2014 (Table 1).

**Table 1:** Number of invasive B stains isolated in Portugal from the first July 2011 to 30^th^ June 2015, and 4CMenB strain coverage predicted by MATS.

Epidemiological Year	N° total of strains	Covered	
		N°	%
2011–12	28	20	71.4
2012–13	23	15	65.2
2013–14	19	14	73.7
2014–15	36	23	63.9
Total	106	72	67.9

Strains covered by one antigen represent 32.1% of the total of isolates, 29.2% of strains were covered by two antigens and 6.6% by three antigens. No strain had all the four antigens (Table 2). Antigens that most contributed for coverage were NHBA and fHbp.

**Table 2:** Percentage of Portuguese strains covered by each one of 4CMenB and combinations.

Antigen Combination	N° of Strain	% of coverage of each antigen or combination
fHbp	16	15.1%
NHBA	15	14.2%
NadA	2	1.9%
PorA	1	0.9%
fHbp+NHBA	18	17.0%
PorA+fHbp	5	4.7%
PorA+NHBA	8	7.5%
PorA+fHbp+NHBA	7	6.6%
No Antigen	34	32.1%

**Fig 1:**
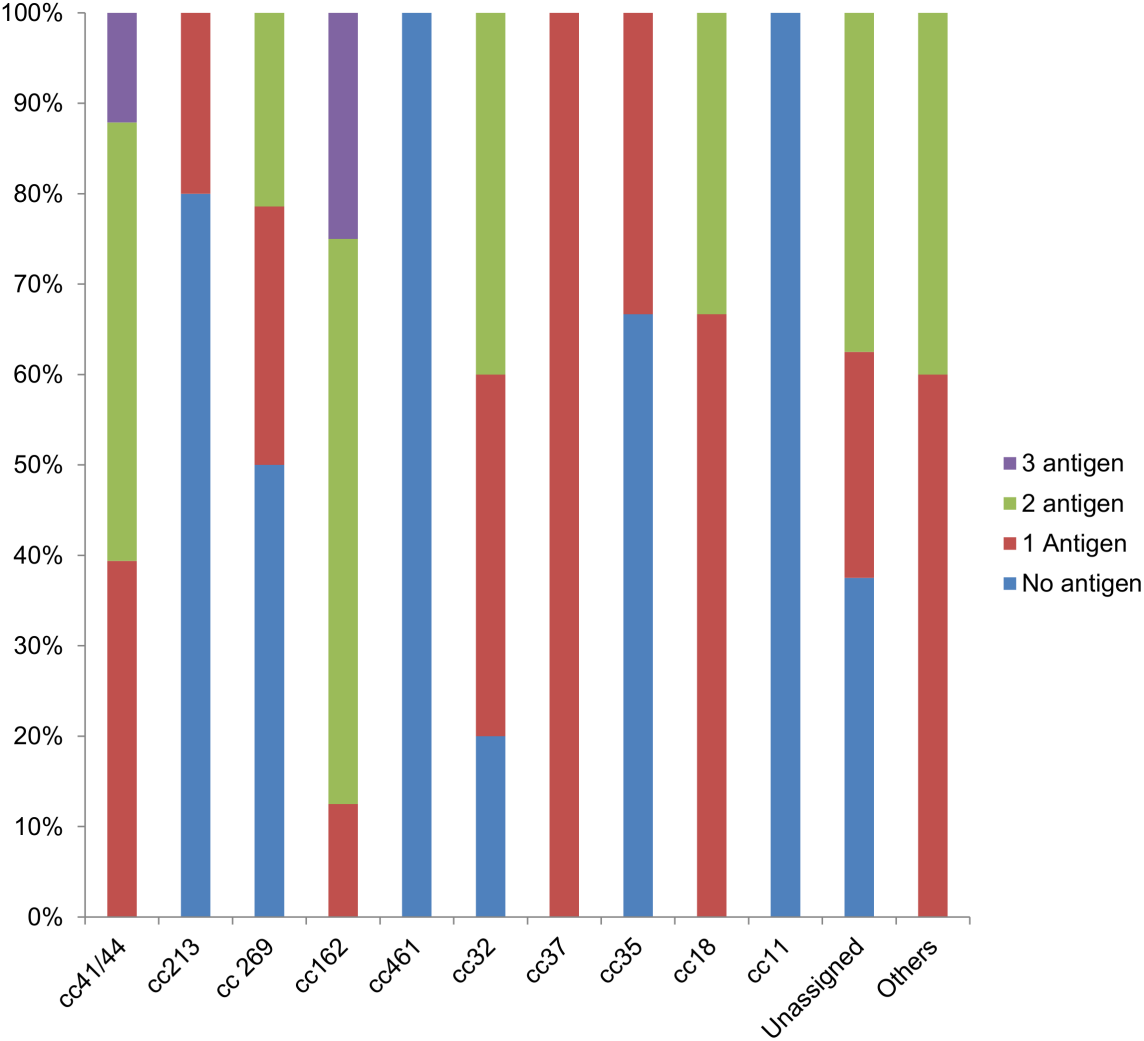
Proportion of strains predicted by MATS to be covered containing one or multiple vaccine antigens and strains not covered, within each clonal complex.

The proportion of strains containing one or multiple vaccine antigens, or containing no antigens, within each clonal complex is shown in Fig 1.

Ninety eight of the studied strains belonged to 15 different clonal complexes (cc) as described in Table 3. The five single isolates belonged to cc22, cc60, cc254, cc1157 and cc865. All MenB cc11 derived from a serogroup C capsular switching (data not shown) and were not covered by any antigen in MATS (Fig 1 and Table 3).

**Table 3:** Clonal complexes of studied strains and percentage of covered strains predicted by MATS.

clonal_complex	Total of studied strains		Covered strains	
	N°	%	N°	%
41/44	33	31.1	33	100.0
213	15	14.2	3	20.0
269	14	13.2	7	50.0
162	8	7.5	8	100.0
461	7	6.6	0	0.0
32	5	4.7	4	80.0
37	3	2.8	3	100.0
35	3	2.8	1	33.7
18	3	2.8	3	100.0
11	2	1.9	0	0.0
Unassigned	8	7.5	5	62.5
Others	5	4.7	5	100.0
Total	106		72	

Others includes clonal complexes that contribute with one isolate which are cc22, cc60, cc254, cc1157 and cc865

**Fig 2:**
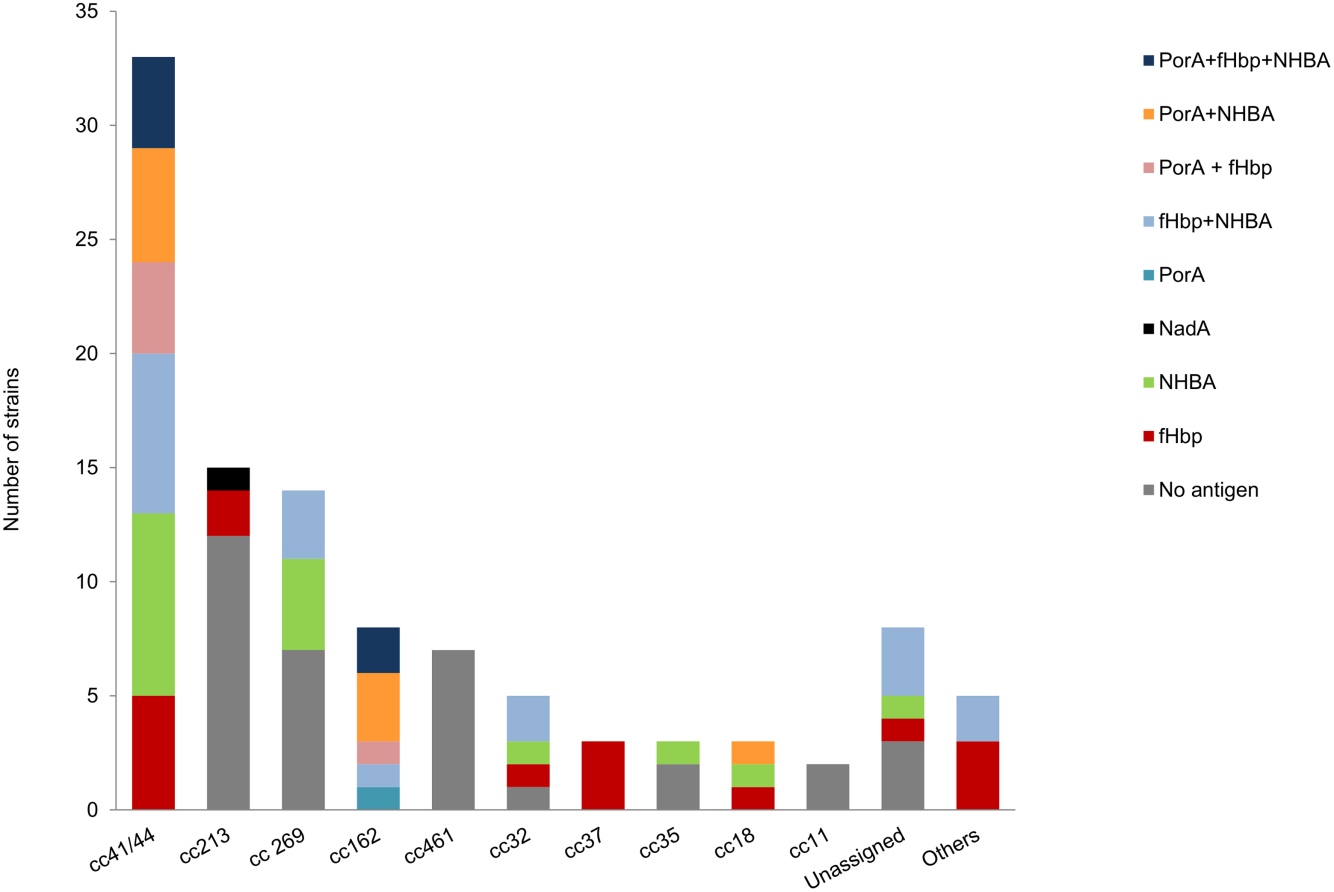
Distribution of the vaccine antigens within each clonal complex.

The vaccine antigens within each clonal complex is shown in Fig 2.

Strain coverage by age group had no significant differences (p-value = 0.939), ranging from 69.7% in infants to 65.2% in over 24 year olds (Table 4). No other demographic factor is noteworthy.

**Table 4:** Percentage of strain coverage predicted by MATS by age group.

Age group	N° total of strains	covered	
		N°	%
< 1 year	33	23	69.7
1–24 years	50	34	68.0
>24 years	23	15	65.2
Total	106	72	67.9

## Conclusions

Strain coverage by 4CMenB was already predicted by MATS in different studies in 12 countries worldwide, counting with 2,679 strains collected from 2006 to 2012, ranging from 66% (95% CI, 43–78%) in Canada to 91% (95% CI, 72–96%) in the United States [14]. In Portugal MATS predicted that 67.9% (95% CI, 56–81%) of invasive B strains isolated in four epidemiological years, from the 1^st^ July 2011 to the 30^th^ June 2015, would be killed by sera of infants vaccinated with 4CMenB, which is in accordance with the MATS predicted strain coverage in five European countries (England and Wales, France, Germany, Italy and Norway) that pointed to 78% coverage (95% CI, 63–90%) for strains collected in the epidemiological year 2007–2008 [15]. However, it is known from a study conducted with UK MenB strains with sera pooled by age group that MATS predicted coverage is lower than coverage measured by human serum bactericidal antibody assay in infants and adolescents vaccinees [16]. It is also observed in a seroprevalence survey conducted after vaccination of students from a U.S. university to control an outbreak, that no case of MenB was reported among vaccinated students although one third of them have not a protective level of bactericidal antibodies against the outbreak strain [17]. This fact can be due to bactericidal antibodies produced lifelong during nasopharyngeal colonization which can be reactive with many bacterial surface proteins.

The lower percentage of vaccine covered strains in the Portuguese study compared to the European study including five countries could be due to the higher proportion of cc213 and cc461 in the studied bacterial population. Effectively, these cc, which together constitute 20.8% of the Portuguese studied strains, vs. 8.8% in the European study are those that less frequently exhibited 4CMenB vaccine antigens [15].

Furthermore, in what concerns the predicted strain coverage, the Spanish study exhibits similarities of the Portuguese: there is 69% of 4CMenB strain coverage in Spain vs. Portugal's 68%; which may be due to the similarity of clonal complex distribution in both countries [18].

MATS predicted coverage of the Portuguese strains represents an important reference information collected by the laboratory before the widespread use of 4CMenB in the country resulting from pediatrician recommendation or, eventually, before its future introduction in the immunization program. It can be expected to observe an increasing proportion of invasive strains not covered, namely cc213 and cc461, but not necessarily the increase in incidence. Considering the proportion of Portuguese strains predicted to be covered by one antigen, which is higher than in other European countries [15,18], genetic characterization of vaccine antigens should be implemented in the reference laboratory in order to detect the emergence of new genotypes that can evade the SBA induced by 4CMenB. MATS must be used not only for monitoring the effectiveness of the vaccine but also in studies focused on meningococcal carriers, needed for a better understanding of the impact of the introduction of 4CMenB vaccine in herd immunity. Since 4CMenB antigens can also be present in other serogroups, the implemented surveillance system is also important to evaluate its impact on MD due to strains of other serogroups [19].

## Supporting information

S1 Table Distribution of genotypes (MLST clonal complexes, ST, PorA and FetA) municipality of patient residence, age, sex and 4CMenB predicted coverage. (TIF)
